# Impact of Ramadan Fasting on Patients with Epilepsy: A Systematic Review

**DOI:** 10.7759/cureus.79157

**Published:** 2025-02-17

**Authors:** Mohamed Mahdaoui, Yahya Naji, Mohamed Chraa, Najib Kissani, Nissrine Louhab

**Affiliations:** 1 Neurology, Mohammed VI University Hospital, Marrakesh, MAR; 2 Neurology, Faculty of Medicine and Pharmacy of Agadir, Ibn Zohr University, Agadir, MAR; 3 Neurology, University Medical Center of Agadir, Agadir, MAR; 4 Neurology, Hopital Razi, Marrakesh, MAR; 5 Medicine and Pharmacy, Cadi Ayyad University, Marrakesh, MAR

**Keywords:** epilepsy, epilepsy education, ketogenic diets, ramadan fasting, risk of seizure

## Abstract

Ramadan fasting, a religious practice observed by Muslims worldwide, involves abstaining from food, drink, and other specified activities from dawn to sunset. For patients with epilepsy (PWE), fasting during Ramadan presents unique challenges due to potential disruptions in medication schedules, changes in sleep patterns, and alterations in diet, which can influence seizure control. This literature review synthesizes current research on the impact of Ramadan fasting on seizure frequency, quality of life (QoL), and physiological changes in PWE. The review also examines existing clinical guidelines for managing epilepsy during Ramadan and identifies significant gaps in the literature that warrant further investigation.

Through a systematic search of three electronic databases, namely PubMed, ScienceDirect, and Google Scholar, relevant studies were identified and analyzed according to the Preferred Reporting Items for Systematic Reviews and Meta-Analyses (PRISMA) guidelines. The review reveals a complex relationship between fasting and epilepsy, with some patients experiencing stable or improved seizure control, while others face increased seizure risk. Medication adherence, sleep disruption, and individual patient characteristics are critical in determining outcomes. The findings underscore the importance of personalized care strategies for PWE who wish to fast during Ramadan, including pre-Ramadan consultations, tailored medication regimens, and ongoing monitoring throughout the fasting period.

Despite the progress made in understanding the effects of Ramadan fasting on epilepsy, significant gaps remain. The review highlights the need for future studies to assess the long-term impact of fasting, research on diverse patient populations, and the development of evidence-based clinical guidelines. Addressing these gaps is essential to improve the management of epilepsy during Ramadan and ensure that patients can safely observe their religious practices while maintaining optimal seizure control.

## Introduction and background

Ramadan is the ninth month of the Islamic calendar, during which healthy pubescent Muslims observe a fasting period lasting up to 30 consecutive days. Fasting involves abstaining from food, drink, sexual activity, and smoking from dawn to sunset. Prepubescent children, menstruating women, and people with certain chronic illnesses are exempt from fasting. Ramadan, the holy month of Islam, involves significant changes to daily routines, including consuming two meals, one before dawn (Suhoor) and the other after sunset (Iftar). Muslims also participate in night prayers, Taraweeh and Tahajjud, during which long portions of the Koran are recited. The Islamic calendar shifts by around 11 days each year compared with the Gregorian calendar, which means that fasting can extend into the late evening in some countries during the summer months. Fasting during Ramadan requires adjustments to lifestyle and circadian rhythms [1]. These changes, combined with altered medication habits, metabolic changes, reduced or fragmented sleep duration, emotional stress, and fatigue, can influence the occurrence of seizures during Ramadan fasting [2].

Epilepsy is a neurological disorder characterized by recurrent, unprovoked seizures that can vary in severity and frequency. Effective management of epilepsy requires strict adherence to anti-seizure medications (ASMs) to maintain stable drug levels and to prevent seizures [3]. The risks associated with Ramadan fasting for patients with epilepsy (PWE) are multiple, including changes in sleep patterns, alterations in diet, and, most critically, disruptions in medication schedules. These factors contribute to an increased risk of seizures, making it essential to understand how Ramadan fasting impacts epilepsy [4].

Intermittent fasting (IF) and Ramadan fasting are two forms of fasting, but they differ in purpose, structure, and flexibility. Intermittent fasting is a health-focused practice used for weight management, improved metabolic health, and overall well-being. It allows for flexibility in fasting schedules. Outside of fasting hours, food and drink, including water, are generally permitted, making IF a versatile and adaptable approach to fasting [5].

On the other hand, Ramadan fasting is a religious obligation observed by Muslims during Ramadan. It is a spiritual practice with strict rules: fasting from dawn until sunset, food, drink, and other specified activities are prohibited [6]. The timing is fixed according to the Islamic lunar calendar, and fasting must be observed daily for the entire month. Unlike IF, Ramadan fasting does not offer flexibility in terms of schedule or practice, as it is deeply rooted in religious traditions. The health implications of fasting during Ramadan are influenced by its rigid structure, including the lack of water during fasting hours and potential changes in diet and sleep patterns [7].

The impact of Ramadan fasting on PWE is multifaceted, shaped by physiological and behavioral factors. While some patients experience improved seizure control, likely due to fasting-induced ketosis, others face increased seizure risks. These adverse outcomes are more pronounced in PWE with underlying metabolic conditions or inconsistent adherence to ASM regimens. Hypoglycemia, dehydration, and disrupted ASM schedules can destabilize neuronal activity, underscoring the need for tailored management to mitigate risks while respecting religious practices [4,7].

This literature review explores existing research on the impact of Ramadan fasting on seizure frequency, quality of life (QoL), and metabolic changes in epilepsy patients. It also discusses current clinical guidelines for the management of epilepsy during Ramadan and identifies gaps in the literature that warrant further study.

This review synthesizes the current research on the relationship between Ramadan fasting and epilepsy. It highlights the challenges and benefits of fasting for PWE and identifies areas for further research. By providing a comprehensive overview of the subject, this review aims to inform clinical practice and guide future research.

## Review

Literature review

We conducted a thorough search of PubMed, ScienceDirect, and Google Scholar to find relevant papers. The search was conducted from the earliest available date to January 2024. Preferred Reporting Items for Systematic Reviews and Meta-Analyses (PRISMA) guidelines were followed, and rigorous standards were used to select studies. The risk of bias in cohort studies was assessed using the Critical Appraisal Skills Programme (CASP) checklist. Articles were included if they explained the study protocol and were written in English. Research on ketogenic diets (KDs) and IF was not included in the analysis.

Published studies on the effects of Ramadan fasting on PWE were searched for. Article titles containing the terms "Ramadan," "fasting," and "epilepsy" were included in the search (Appendix A). In addition, publications were searched in the bibliographies of the retrieved studies. A description of the study technique, publication in English, and exclusion of studies on KDs and IF.

**Figure 1:**
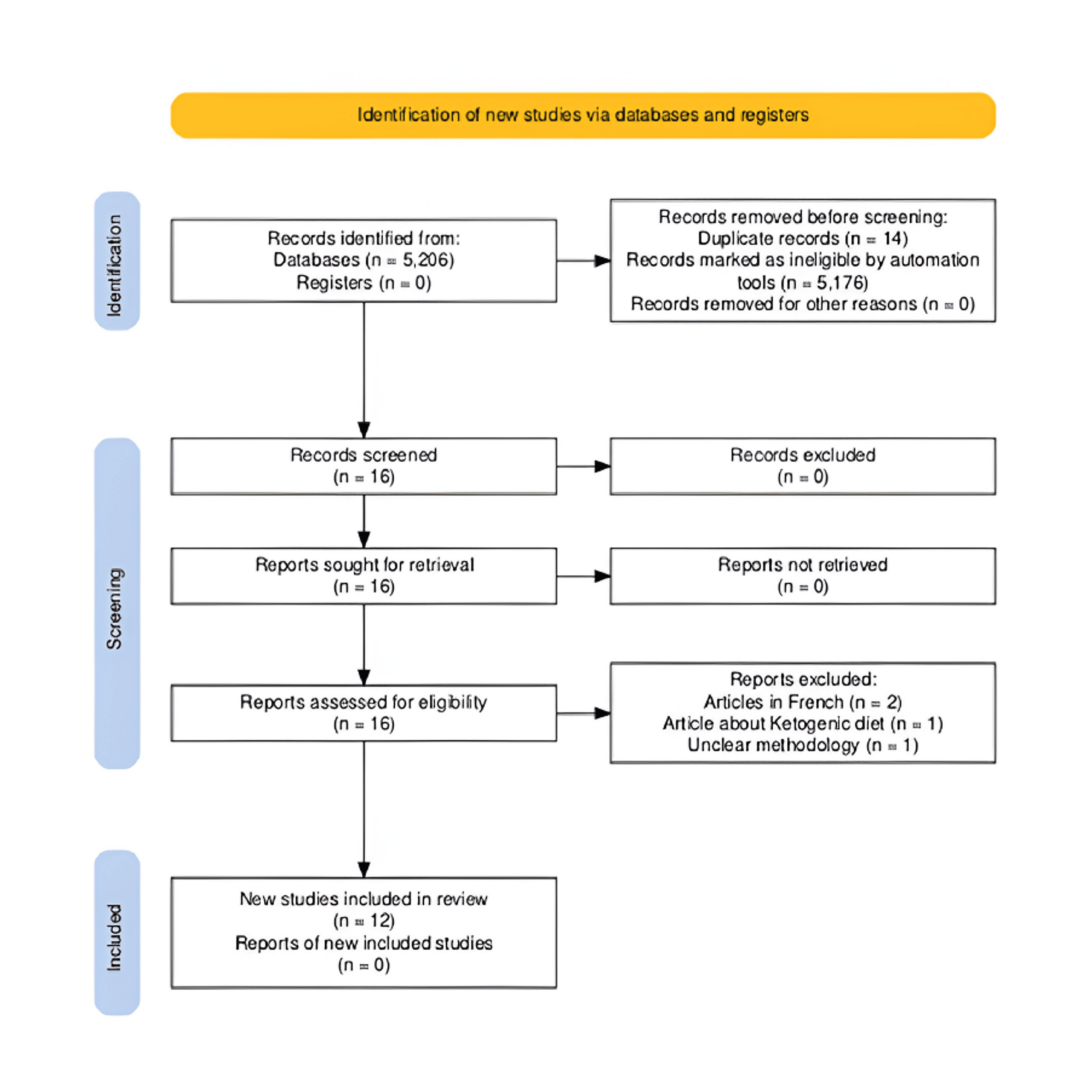
A PRISMA flow diagram outlining the search strategy for a literature review of the effects of Ramadan fasting on PWE PRISMA: Preferred Reporting Items for Systematic Reviews and Meta-Analysis; PWE: patients with epilepsy

Two researchers independently reviewed the abstracts and titles to determine whether the papers met the requirements. When in doubt, the full papers were reviewed by both researchers, and the authors met to reach a consensus on final inclusion. The search was limited to peer-reviewed journal articles and abstracts without regard to publication date. The PRISMA flowchart outlines the systematic process of article selection (Figure 1). Through the search, 5,206 records were identified from databases (PubMed, ScienceDirect, Google Scholar), with 16 screened after eliminating ineligible papers by automation tools and duplicate removal. After title/abstract screening, 16 full-text articles were assessed for eligibility, and four were excluded due to irrelevance, non-English language, or failure to meet inclusion criteria (e.g., studies on KDs or IM were excluded). Ultimately, 12 articles met the inclusion criteria, focusing on the impact of Ramadan fasting on epilepsy, and were included in the review.

Physiological impact of fasting on epilepsy

Metabolic Changes During Fasting

Fasting during Ramadan induces significant metabolic changes that have important implications for PWE. The body shifts from using glucose as the primary energy source to an increased reliance on fatty acids and ketone bodies during fasting. This metabolic shift may affect brain function and, in some cases, seizure activity. Akıncı et al. studied these metabolic changes in PWE during Ramadan fasting. The study highlighted that fluctuations in blood glucose levels and electrolyte imbalances could lower the seizure threshold and increase the risk of seizures [8].

Hypoglycemia, a known seizure trigger, is a concern during prolonged fasting, especially in patients with poorly controlled epilepsy [8,9]. In addition, the authors noted that dehydration, which is common during Ramadan due to reduced fluid intake, can lead to electrolyte disturbances such as hyponatremia or hypernatremia, which may exacerbate seizures, especially in patients who are sensitive to changes in sodium levels. These findings highlight the importance of close monitoring of metabolic parameters in PWE during Ramadan, especially in those with a history of metabolic instability [8].

Khattab et al. reported that glucose, sodium, and potassium levels remained within normal ranges, suggesting that fasting did not adversely affect seizure control in these PWE [10].

In addition, Ramadan fasting may affect other physiological factors such as circadian rhythms. Disrupted sleep patterns during Ramadan, often due to late-night meals (Suhoor) and early morning prayers (Fajr), can lead to sleep deprivation, a well-documented seizure precipitant [1].

Meimand et al. provided insight into the potential benefits of a high-fat, high-protein diet during Ramadan, similar to the KD. The KD is known for its anticonvulsant effects, primarily due to its ability to alter glucose metabolism in the brain. The study observed a 40% reduction in emergency room (ER) visits due to epilepsy during Ramadan, attributed to dietary changes and improved medication adherence. These findings suggest that the metabolic effects of fasting, combined with a KD-like diet, may improve seizure control during Ramadan [11,12].

Despite these potential benefits, it is important to note that the mechanisms underlying the effects of fasting and KD on epilepsy are different. While fasting increases the rate of inhibition of neuronal activity, KD may have an antiepileptic effect by reducing the production of epileptic discharges [11]. The combination of these two effects during Ramadan may synergistically enhance the overall antiepileptic effect, although further research is needed to fully understand these mechanisms and their implications for epilepsy management during Ramadan [12].

The implications of these physiological changes are profound, suggesting that healthcare providers need to be vigilant in monitoring and managing PWE who choose to fast during Ramadan. This includes regular blood testing to check glucose and electrolyte levels and advice on maintaining hydration and adequate nutrition during non-fasting hours. Further research is warranted to explore these mechanisms and their implications for epilepsy management during Ramadan to ensure that patients can safely observe their religious practices.

Medication Adherence and Fasting

Effective treatment of epilepsy relies on the consistent administration of ASMs. These medications must be taken at specific intervals to maintain therapeutic drug levels in the bloodstream and prevent seizures. Ramadan fasting disrupts normal eating and medication schedules, making it challenging to maintain consistent ASM levels [13].

Mahmoud et al. provide comprehensive guidance on adjusting ASM regimens during Ramadan, suggesting that patients and healthcare providers collaborate to develop individualized medication schedules that align with fasting hours. For instance, ASMs typically taken three times a day may need to be adjusted to twice a day, with doses administered at a pre-dawn meal (Suhoor) and a post-sunset meal (Iftar). However, such adjustments must be made cautiously, as altering the dosing schedule may impact the pharmacokinetics of the drug and potentially reduce its efficacy [14].

A study by Khattab et al. found that epileptic patients taking continuous doses of carbamazepine (CBZ) twice daily could safely fast during Ramadan without significant fluctuations in serum drug concentrations. The study found no significant differences in CBZ concentration before and during Ramadan, suggesting that adjusting ASM regimens to a twice-daily schedule could maintain effective seizure control [10].

A major concern during Ramadan is the risk of medication non-adherence. Some patients may skip doses to avoid breaking their fast, or they may inadvertently miss doses due to a change in daily routine. Nonadherence can lead to subtherapeutic drug levels, increasing the risk of breakthrough seizures [11].

Mahmoud et al. emphasized the importance of pre-Ramadan consultations, where healthcare providers can assess patients' current seizure control, review their ASM regimen, and provide tailored advice on managing their condition during Ramadan. In addition to adjusting medication schedules, healthcare providers must educate patients about the importance of adherence and the risks of nonadherence, advising them to take medications with adequate fluids during non-fasting hours and to avoid skipping doses even if they feel well [14].

The relationship between fasting, medication adherence, and seizure control is complex and requires careful management. Healthcare providers must balance the religious and cultural significance of fasting with the medical necessity of maintaining consistent ASM levels [14]. This may include providing patients with strategies to mitigate the risks associated with fasting, such as using long-acting ASMs or adjusting the timing of doses to minimize fluctuations in drug levels [11].

Meimand et al. highlighted a reduction in emergency department visits for epilepsy during Ramadan, attributed in part to improved medication adherence facilitated by reduced travel and more consistent routines during the holy month. However, the potential for non-adherence due to fasting-related challenges remains a critical concern [11].

Seizure frequency during Ramadan fasting

Changes in Seizure Frequency

The impact of Ramadan fasting on seizure frequency has recently become a focal point of research. Several studies have explored whether fasting during Ramadan increases or decreases the likelihood of seizures in PWE, producing varying results.

Gomceli et al. reported that fasting during Ramadan is associated with a statistically significant increase in seizure frequency. This risk appeared higher among patients who altered their medication regimens compared to those who did not. No significant difference in seizure frequency during Ramadan was noted between patients undergoing monotherapy and those on polytherapy [15]. Similarly, Mahdaoui et al. suggested that Ramadan fasting could exacerbate epilepsy, particularly in patients who have recently changed their treatment regimen or are suffering from sleep deprivation [16].

AL-Mahdawi's publication in 2002 was the first to explore this topic. This study lasted three years and revealed that over 50% of participants successfully fasted during Ramadan. The researchers concluded that PWEs who have normal neurological exams and electroencephalography results are more likely to tolerate fasting than other patients [17].

Magdy et al. conducted a prospective observational study investigating seizure frequency in PWE during Ramadan, as well as the month before (Shaaban) and the month after (Shawwal). This study involved 321 patients with various types of epilepsy and aimed to determine whether fasting had a significant impact on seizure activity. The findings showed that Ramadan fasting was associated with a notable reduction in the frequency of focal and myoclonic seizures during and after Ramadan. However, the study did not observe significant changes in the frequency of generalized tonic-clonic seizures [4].

The reduction in focal and myoclonic seizures during Ramadan can be attributed to several factors. First, metabolic changes induced by fasting, such as increased ketone body production, may stabilize neuronal activity in some patients. Second, the structured Ramadan routine, with specific times for eating, praying, and resting, may enhance seizure control in certain individuals. This consistency could reduce stress and anxiety, known seizure triggers, thus improving overall seizure control [4].

However, Magdy et al. also noted that while some patients experienced a decrease in seizure frequency, others reported an increase in seizures during Ramadan. This variability suggests that fasting's effect on seizure frequency is highly individualized and may depend on factors like the type of epilepsy, the patient's baseline seizure control, and adherence to ASMs during Ramadan. Post-fasting effects, as suggested by Magdy et al., demonstrated a significant improvement in focal and myoclonic seizures in the Shawwal group compared to the Shaaban group [4]. Alqadi et al. conducted a multilinear regression analysis and observed a significant 29% reduction in seizures during the post-fasting period compared to the baseline [13].

In Meimand’s study, researchers noted a significant decrease in ER visits due to epilepsy during Ramadan, which was sustained even in the following month. This reduction in seizure frequency was attributed to several factors, including dietary changes, improved medication adherence, and the overall peaceful environment during Ramadan. However, the study also noted an increase in seizure frequency in the month after Ramadan, though not to the levels observed before Ramadan. This implies that while fasting effects may extend beyond Ramadan, they might not be sustained long-term [11].

Additionally, the risk of seizure recurrence due to ASM discontinuation is a significant concern. Discontinuation of antiepileptic drugs often stems from intolerance or side effects, which can profoundly impact the quality of life for PWE. Authors reported recurrence rates of epileptic attacks ranging from 23% to 63% following ASM discontinuation. Thus, careful management and patient education are essential to ensure adherence to ASM regimens during Ramadan, minimizing the risk of exacerbated seizures [11].

Darwish et al. conducted a prospective cohort study on epileptic adolescents during Ramadan, finding no significant difference in seizure frequency during Ramadan compared to the month before, but a significant reduction in seizure severity. This indicates that while fasting may not significantly alter seizure frequency, it may positively impact severity for some patients. The study also found that uncontrolled epileptic patients experienced a significant reduction in both seizure frequency and severity during Ramadan, suggesting that fasting could be beneficial for seizure control in this subgroup [18].

Predictors of Successful Fasting

Identifying factors that contribute to successful fasting without increased seizure risk is crucial for patient management. Pre-Ramadan seizure control, stable ASM regimens, and consistent lifestyle factors, such as adequate sleep, serve as key predictors of successful fasting. Patients with well-controlled epilepsy and a stable medication routine are more likely to fast safely. In contrast, those with uncontrolled seizures or frequent medication adjustments may face higher risks. This underscores the importance of thorough pre-Ramadan assessments and personalized care plans.

Magdy et al.identified several key factors associated with successful fasting, defined as the ability to fast during Ramadan without an increase in seizure frequency. These include good seizure control before Ramadan, stable antiepileptic drug regimens, and adequate sleep [19].

Patients with well-controlled epilepsy before Ramadan were more likely to fast successfully, suggesting that those with fewer seizures at baseline have a lower risk of experiencing breakthrough seizures during fasting. Stable ASM regimens also played a significant role, with patients who adhered consistently to their medication schedules experiencing fewer seizures. This finding underscores the importance of medication adherence, even in the face of the challenges posed by fasting.

Sleep patterns also emerged as a significant predictor of fasting success. Patients who maintained adequate sleep during Ramadan were less likely to experience seizures. This is particularly important given the disruption of sleep patterns during Ramadan, with late-night prayers (Taraweeh) and early-morning meals (Suhoor) potentially reducing the overall duration and quality of sleep. Sleep deprivation is a well-known seizure trigger, and maintaining good sleep hygiene during Ramadan is essential for reducing seizure risk [19].

The identification of these predictors can help healthcare providers assess which patients are likely to fast successfully and which may require additional support or modifications to their fasting practices. For example, patients with poorly controlled epilepsy may need to be advised against fasting or may require more frequent monitoring during Ramadan [19].

Fathalla et al. explored the feasibility of fasting in children with epilepsy, finding that with proper physician guidance and medication compliance, fasting during Ramadan was generally safe. The study observed only two seizures in 19 fasting cycles among pediatric patients, all of whom reported adherence to their prescribed medication schedules. They suggested that PWEs with a history of controlled epilepsy, ASM monotherapy, and a classification of partial seizures have a low risk for seizure occurrence during Ramadan fasting [20].

Khattab et al. further supported the idea that stable ASM regimens and careful monitoring are key to successful fasting. By maintaining stable serum concentrations of CBZ, patients were able to fast without experiencing significant fluctuations in seizure control. This suggests that with proper adjustments to medication schedules, fasting can be safely managed in many epilepsy patients [10].

Quality of life and psychological impact

Impact on QoL

Quality of life is a critical consideration for PWE during Ramadan. While seizure control is often the primary focus, it is equally important to consider how fasting affects the overall well-being of these patients. Quality of life encompasses various dimensions, including physical health, psychological well-being, social functioning, and the ability to perform daily activities [21].

Alqadi et al. conducted a study to assess the impact of Ramadan fasting on QoL in PWE, utilizing standardized QoL questionnaires to evaluate changes in physical health, mental health, and social functioning during Ramadan. Interestingly, although the study found a significant reduction in seizure frequency during Ramadan, there were no significant improvements in QoL measures [13].

Several factors could explain this discrepancy between seizure control and QoL. First, the physical demands of fasting, such as fatigue, dehydration, and altered sleep patterns, counteract any potential benefits of reduced seizure frequency. Even if patients experience fewer seizures, the overall strain of fasting on the body could negatively impact their QoL [22].

Second, the psychological stress associated with fasting and managing epilepsy during Ramadan may also play a role. The fear of having a seizure while fasting, the pressure to adhere to religious practices, and concerns about medication adherence can contribute to increased anxiety and reduced QoL. For some patients, the anticipation of potential health risks during Ramadan may outweigh the actual physical benefits of fasting [13,23].

Finally, social and cultural factors may influence QoL during Ramadan. The communal aspects of Ramadan, such as breaking the fast with family and attending religious gatherings, are significant for many Muslims. However, PWE may feel excluded or stigmatized if they are unable to fully participate in these activities due to their condition. This social isolation can negatively impact their QoL [13].

The findings of Alqadi et al. highlight the need for a holistic approach to managing epilepsy during Ramadan. Healthcare providers should not only focus on seizure control but also consider the broader impact of fasting on patients' physical and psychological well-being. This may involve offering support for managing fatigue, stress, and social challenges during Ramadan [13].

Psychosocial and Cultural Factors

The decision to fast during Ramadan is deeply rooted in religious and cultural beliefs. For many Muslims, fasting is a fundamental aspect of their faith, and the desire to participate fully in Ramadan is strong, even in the face of health challenges. This cultural context can influence how PWE approach fasting and their willingness to follow medical advice.

For some patients, the religious significance of Ramadan may compel them to fast despite potential risks. They may feel that fasting is a religious obligation and that skipping it could have spiritual consequences. This belief can sometimes lead patients to disregard medical advice or downplay the severity of their condition, thereby increasing the risk of adverse health outcomes during Ramadan [23].

A study by Fathalla et al. emphasized the importance of culturally sensitive care in managing epilepsy during Ramadan. The study found that many patients were willing to adjust their fasting practices based on physician guidance, provided the advice was delivered in a culturally respectful manner. This underscores the need for healthcare providers to engage in open, empathetic communication with patients, helping them to make informed decisions that align with both their health needs and religious beliefs [20].

Conversely, some patients may experience guilt or shame if they are advised not to fast due to their epilepsy. They may feel that they are failing in their religious duties or letting down their community. These feelings can lead to isolation, anxiety, and depression, further complicating the management of their condition during Ramadan [6].

Healthcare providers must be sensitive to these psychosocial and cultural factors when advising PWE about fasting. It is essential to have open and empathetic discussions that respect the patient's religious beliefs while also addressing the medical risks associated with fasting. Providers should emphasize that Islam allows exemptions from fasting for individuals with chronic illnesses and that health is a priority in the faith [6].

In some cases, a collaborative approach involving religious leaders may be beneficial. Religious leaders can offer spiritual guidance and reassurance to patients, helping them understand that their health needs align with their religious obligations. This collaboration can alleviate the psychological burden of not fasting and encourage patients to follow medical advice without feeling conflicted about their religious duties [23].

Clinical guidelines and recommendations

Existing Guidelines for Managing Epilepsy During Ramadan

To support healthcare providers in managing epilepsy during Ramadan, there is a significant need for comprehensive clinical guidelines. These guidelines are essential for offering recommendations on adjusting ASM regimens, monitoring patients, and providing pre-Ramadan counseling.

Mahmoud et al. offer detailed guidance on managing epilepsy during Ramadan. Their recommendations include assessing each patient's seizure control and medication adherence before Ramadan, adjusting ASM regimens to align with the fasting schedule, and educating patients on the importance of maintaining hydration and adequate nutrition during non-fasting hours. They also suggest regular follow-ups during Ramadan to monitor seizure activity and address any emerging issues [14].

These guidelines emphasize the importance of individualized care, recognizing that each patient has unique needs. The ability to fast safely depends on factors such as the type of epilepsy, seizure frequency, and overall health. By tailoring the management plan to each patient, healthcare providers can help minimize the risks associated with fasting.

Khattab et al. underscore the need for ongoing monitoring and support during Ramadan, particularly for patients who may face challenges in adhering to their medication regimens. The study recommends that healthcare providers collaborate closely with patients to develop individualized fasting plans that minimize the risk of seizure exacerbation while allowing patients to observe their religious practices [10].

Gaps in Guidelines and Areas for Improvement

Despite the availability of some guidelines, significant gaps persist in the management of epilepsy during Ramadan. Existing guidelines are often rooted in expert opinion and clinical experience rather than strong empirical evidence, underscoring the need for additional research to inform guidelines and provide evidence-based recommendations.

One area where guidelines could improve is in managing patients with poorly controlled epilepsy. Current guidelines frequently focus on patients with stable epilepsy who are already well-controlled on their ASMs. However, patients with more severe or refractory epilepsy may require different strategies, such as more frequent monitoring, alternative medication regimens, or even advice to avoid fasting altogether [14].

Another gap in the guidelines is the lack of consideration for the diversity of patient experiences. Patients with different types of seizures, comorbid conditions, or cultural backgrounds may have unique needs during Ramadan. Future guidelines should strive to be more inclusive, providing tailored recommendations for various subgroups of patients [19].

Magdy et al. emphasize the necessity for more research on the impact of fasting on different seizure types, as well as the effects of specific ASMs during Ramadan. The study calls for larger, more diverse studies to establish a stronger evidence base for clinical guidelines. Additionally, there is a need for guidelines that address the psychosocial and cultural factors influencing patients' decisions to fast, enabling healthcare providers to offer more comprehensive, patient-centered care [4].

Finally, guidelines should address the psychological and social aspects of fasting for PWE. While current guidelines primarily focus on medical management, they should also include recommendations for supporting patients' mental health and tackling the social challenges they may face during Ramadan. This could involve providing resources for stress management, promoting social support networks, and encouraging a holistic approach to care.

Gaps in the literature and future research directions

Limitations of Current Studies

The studies conducted on the impact of Ramadan fasting on epilepsy, while informative, often have limitations that affect their generalizability and reliability. One of the most significant limitations is the small sample sizes in many of these studies. Such sizes reduce the statistical power of a study, making it challenging to detect significant differences or effects and draw definitive conclusions.

Another limitation is the absence of control groups in most studies. Given the ethical considerations involved, it is impractical to ask individuals who plan to fast for religious reasons to refrain from doing so solely for research purposes. However, this limitation could be mitigated by incorporating a control group of individuals who do not intend to fast. Without such a control group, attributing changes in seizure frequency or other outcomes specifically to fasting becomes challenging. Including control groups in future research would strengthen the evidence base and allow for more robust comparisons. Khattab et al. noted that the lack of control groups in many studies complicates drawing firm conclusions about the effects of fasting on epilepsy, emphasizing the need for more rigorous research designs [10].

A common limitation is the reliance on self-reported data. Many studies depend on patients' self-reports of seizure frequency, medication adherence, and QoL. While self-reports are valuable, they are susceptible to biases, such as recall bias or social desirability bias. Objective measures, such as electronic seizure diaries or blood tests to monitor drug levels, could yield more accurate and reliable data.

Finally, most studies are conducted over a short duration, typically limited to the Ramadan period or the immediate months before and after. Longitudinal studies that follow patients over multiple years are necessary to assess the long-term impact of Ramadan fasting on PWE, including any cumulative effects on seizure control, medication efficacy, and overall health.

Suggested Areas for Future Research

Future research should prioritize longitudinal studies to assess the long-term effects of Ramadan fasting on epilepsy, examining variations in seizure types and patient demographics. Research should also investigate the mechanisms underlying the observed benefits of fasting in some patients, such as potential similarities between fasting-induced metabolic states and KDs. Additionally, studies should focus on creating more accurate predictive models for fasting success to refine clinical guidelines and enhance patient outcomes.

There is a clear need for more research on the long-term effects of Ramadan fasting on epilepsy, particularly how fasting impacts different seizure types and patient populations. Longitudinal studies that track patients through multiple fasting periods could provide valuable insights into the cumulative effects of fasting on seizure control and overall health outcomes.

It is crucial to investigate the mechanisms behind the observed benefits of fasting in certain patients. Understanding how fasting-induced metabolic states compare to those achieved through KDs could pave the way for innovative approaches to managing epilepsy during Ramadan.

Table 1 summarizes all the studies which were included in this literature review.

**Table 1:** A comparative analysis of all the studies included in the review EEG: electroencephalogram; CBZ: carbamazepine; ASM: anti-seizure medication; ER: emergency room

Author(s), year, and place		Type of study	Key findings
Al Mahdawi.,1995 [17], Bagdad, Iraq		Prospective observational study; Recruited 35 participants	The patients who did not observe the Ramadan fasting were classified as 'failure to fast during Ramadan,' while those who were able to successfully continue the Ramadan fasting were classified as 'succeeded to fast during Ramadan'. The most favorable outcomes were observed in patients with normal neurological examinations and normal EEG records before the fasting period [17].
Gomceli et al., 2008 [15], Ankara, Turkey		Prospective observational study; Recruited 114 participants	In patients diagnosed with epilepsy, there was a noted increase in seizure frequency, despite their consistent adherence to prescribed drug regimens. Various factors, including changes in drug intake patterns, reduction in sleep duration and/or sleep fragmentation, emotional stress, and fatigue, either individually or collectively, have an impact on the occurrence of seizures during Ramadan fasting [15].
Khattab et al., 2008 [10], Mosul, Iraq		A case series study; Recruited 40 participants	During Ramadan, the same total daily CBZ dose was administered twice daily instead of three times. Blood tests before and during Ramadan showed no significant changes in CBZ levels, glucose, sodium, or potassium. The study concluded that well-controlled epileptic patients can safely fast Ramadan while maintaining their CBZ regimen twice daily without compromising seizure control or metabolic stability [10].
Akinci et al., 2012 [8], Ankara, Turkey		Prospective observational study; Recruited 40 participants	No significant difference was observed in the metabolic state between the fasting group and the control group. However, a notable disparity was identified in the fasting group of PWE, which exhibited higher levels of ketones compared to the control group [8].
Fathalla, 2014 [20], Abu Dhabi, UAE		Prospective observational study; Recruited 20 participants	This study aimed to observe seizure occurrence during Ramadan among fasting Muslim children with epilepsy on ASMs. Only two seizures were reported across 19 fasting cycles, with no adverse effects from medication schedule changes. The study concluded that fasting during Ramadan is feasible for children with epilepsy under proper physician guidance and patient compliance, emphasizing the importance of anticipatory guidance for managing risks during religious activities [20].
Alqadi et aL, 2020 [13], Riyadh, Saudi Arabia		Prospective observational study; Recruited 37 participants	Ramadan fasting could potentially have a beneficial influence on seizure management in PWE, with its effects persisting even beyond the fasting period. Consequently, the fasting practice does not appear to significantly impact the scores indicating quality of life [13].
Magdy et al., 2020 [4], Cairo, Egypt		Prospective observational study; Recruited 321 participants	This study serves to validate the beneficial nature of Ramadan fasting for individuals of the Muslim faith who are affected by active epilepsy, specifically those experiencing focal, myoclonic seizures, or absence seizures. The observed improvements are not only limited to the duration of the fasting period but also extend beyond the conclusion of Ramadan (known as the post-fasting effect) [4].
Magdy et al., 2020 [19], Cairo, Egypt		Prospective observational study; Recruited 430 participants	They recommend ensuring effective seizure control and sufficient sleep duration. Each additional week of being seizure-free before Ramadan, as well as each additional hour of sleep, was found to be associated with a 10% and 30% increase in the likelihood of successfully observing the Ramadan fast, respectively [19].
Mahmoud et al., 2020 [14], Glasgow, Scotland		Review	The risk of experiencing seizures is increased during fasting periods, and the typical medication regimens often need to be adjusted during Ramadan. There is a lack of specific guidance available for healthcare professionals and patients regarding epilepsy and fasting. To address this gap, we suggest categorizing patients into low- and high-risk groups based on the characteristics of their epilepsy and the probability of seizure recurrence [14].
Darwish., 2020 [18], Tanta, Egypt		Prospective observational study; Recruited 56 participants	Comparing seizure frequency and severity during Ramadan to the month before, no significant difference in seizure frequency was found, but seizure severity significantly decreased. Uncontrolled patients showed significant reductions in both frequency and severity during Ramadan. The study concluded that Ramadan fasting is safe for epileptic adolescents, does not increase seizure risk with proper medication compliance, and may improve seizure control in uncontrolled epilepsy [18].
Ebrahimi et al., 2023 [11], Kerman, Iran		Prospective observational study; Recruited 156 participants	This study showed a 40% reduction in epileptic attack-related ER visits during Ramadan, attributed to factors such as dietary changes (high-fat, high-protein meals, and reduced food intake), better medication management, reduced travel, and the calming effects of the holy month. The findings suggest that fasting and lifestyle changes during Ramadan may help control epileptic seizures [11].
Mahdaoui et al., 2023 [16], Marrakech, Morocco		A case series study Recruited 120 participants	Using a structured questionnaire, 51.7% reported disease worsening during Ramadan, primarily due to lack of sleep and sleep fragmentation. Patients who altered their antiepileptic drug regimen were more likely to experience worsening (80%) compared to those who did not. The study concluded that Ramadan fasting may aggravate epilepsy, particularly in patients with recent treatment changes, and highlighted the need for future research to develop guidelines for managing epilepsy during Ramadan [16].

## Conclusions

Ramadan fasting presents both challenges and opportunities for PWE, with effects on seizure frequency varying between individuals. While fasting may stabilize neuronal activity for some, risks like medication non-adherence and sleep disruption exist. Healthcare providers should offer personalized care, including pre-Ramadan consultations, tailored medication schedules, and ongoing support, to help patients safely observe Ramadan. Future research should address gaps in understanding long-term outcomes and diverse patient needs, enabling the development of evidence-based guidelines. Clinicians must balance religious observance with health needs, ensuring safe and fulfilling Ramadan experiences for PWE.

## Appendices

Appendix A 

**Table 2:** Search criteria for the literature review

Electronic databases	Search terms
PubMed, ScienceDirect, and Google Scholar	“epilepsy” (AND) “ramadan fasting” ({epilepsy (MeSH Terms)} AND {ramadan fasting (MeSH Terms)} AND {english (la)})
